# Uncertainty, shock and anger: Recent loss experiences of first‐wave COVID‐19 pandemic in Italy

**DOI:** 10.1002/casp.2604

**Published:** 2022-02-13

**Authors:** Sabrina Cipolletta, Lorenza Entilli, Sara Filisetti

**Affiliations:** ^1^ Department of General Psychology University of Padua Padua Italy

**Keywords:** bereavement, community, corona virus, COVID‐19, death, health, pandemic, qualitative analysis, social support

## Abstract

The aim of this study is to explore qualitatively bereavement experiences of family members who have lost a significant other to coronavirus disease 2019 (COVID‐19) in relation to mourners' needs and resources. Twenty individuals bereaved by the first wave of COVID‐19 from the most heavily impacted Italian region were interviewed via video call between 1 and 3 months after their loss. Through a thematic analysis, four main themes were identified: reconstructions of the loss experience, responses to grief, resources and looking forward. The suddenness of the death and lack of farewell by means of a funeral prevented participants from realizing the loss and undertaking a meaning‐making process. When anger was the main reaction to the loss, mourners focussed all their attention on denouncing medical and government institutions. Acceptance occurred particularly in those who found a way to share their grief and use it as a turning point. Participants relied mainly on informal support offered virtually, but mourners may have sought out in‐person comfort in the long term. The results of this study propose new insights for COVID‐19 bereavement support and trace the path for health promotion within a community shook by a communal grief experience. Please refer to the Supplementary Material section to find this article's Community and Social Impact Statement

## 1 INTRODUCTION

Losing a loved person entails sorrow, sadness, hopelessness and disrupted social functioning (Bonanno & Kaltman, 2001). Over time, the bereaved is usually able to pass certain milestones such as learning to manage painful emotions, recreate or strengthen relationships and restore a sense of purpose (Goveas & Shear, 2020). According to the meaning making model, mourners engage in three major activities to reconstruct meaning in response to loss: sense making, benefit finding and identity change (Neimeyer, 2001). Adaptation to loss, therefore, requires mourners to be able to construct a new reality in which their core beliefs have new meaning (O'Connor, 2003).

However, this process can have very different outcomes when traumatic deaths are involved (Kokou‐Kpolou, Fernández‐Alcántara, & Cénat, 2020). A traumatic death is a death occurred in a sudden, violent or unexpected condition, such as in suicides, homicides and natural disasters (Kristensen, Weisæth, & Heir, 2012). The literature has outlined how the traumatic circumstances of a loved one's death may negatively impact the bereavement process (Cipolletta, Entilli, Bettio, & De Leo, 2021; Kristensen et al., 2012; Neimeyer & Burke, 2017): reactions to traumatic deaths usually involve rumination, avoidance, an intense yearning to be with the loved one and, most of all, difficulty to accept the loss (Howarth, 2011).

During the COVID‐19 pandemic, many individuals, if not entire families, were touched by a death that occurred in unprecedented and violent conditions. Italy was the first Western country touched by the spread of COVID‐19 (Berardi et al., 2020; Marazziti, Pozza, Di Giuseppe, & Conversano, 2020). According to the Italian National Institute of Health, up to March, deaths have exceeded 120,000 in Italy and surpassed 32,000 in the Lombardy region alone – one of the largest and most serious clusters of COVID‐19 in the world (Livingston & Bucher, 2020). Several researchers (Boelen, Eisma, Smid, & Lenferink, 2020; Carr, Boerner, & Moorman, 2020; Eisma, Boelen, & Lenferink, 2020; Gesi et al., 2020; Goveas & Shear, 2020; Johns, Blackburn, & McAuliffe, 2020) have anticipated the high risk of experiencing grief complications due to the unique nature of COVID‐19 bereavements: missing farewell, death perceived as unjust or preventable, witnessing the death and/or suffering or imagining so (i.e., a death that occurred in an ICU or hospital). According to Carr et al. (2020), COVID‐19 deaths fall within the category of ‘bad deaths’ due to the specific conditions in which they take place: pain or physical discomfort (e.g., difficulties breathing, intubation), social isolation from loved ones, psychological distress, lack of awareness or preparation, undignified and hasty treatment and denial of medical treatments (Krikorian, Maldonado, & Pastrana, 2020).

A recently proposed model of uncertainty distress poses the ‘unknown‐ness’, which characterizes the experience of COVID‐19 losses, as the main cause of distress in mourners (Freeston, Tiplady, Mawn, Bottesi, & Thwaites, 2020). COVID‐19 deaths can be classified as ambiguous losses (Testoni et al., 2021) because the death occurs rapidly, unexpectedly for some, and farewell often must be made without the physical presence of the body (Bertuccio & Runion, 2020).

In addition to the generalized uncertainty characterizing the first months of the pandemic (Cipolletta & Ortu, 2021; Marazziti et al., 2020), social distancing requirements and confinement to the home may have hindered grieving people from mourning their loved ones in traditional ways, preventing them from accepting the reality of their loss (Wallace, Wladkowski, Gibson, & White, 2020). Social restrictions have also impacted mourners' support seeking (Parisi, Lagomarsino, Rania, & Coppola, 2021): lower perceived support within the family or social network predicts greater distress, perhaps especially for grievers in need of practical and instrumental support (Carr et al., 2020). All these conditions represent important primary and secondary stressors that increase the risk of negative outcomes in bereavement (Eisma et al., 2020). While it should not be assumed that all COVID‐19 mourners are bound to develop complicated grief, it is important to understand how the extreme circumstances of this loss may open the way for complicated grief in order to identify risk and protective factors (Wallace et al., 2020).

The aim of the present study was to explore qualitatively the experience of bereavement on the part of family members whose loved ones died due to COVID‐19 during the pandemic's first wave in the first and most affected region in a Western country (Lombardy, Italy) and to differentiate among mourners' responses to the loss in relation to their needs and resources. The research questions that guided the study are: how do COVID‐19 mourners make sense of their loss and the events that surround it? How do they look for support in the context of the pandemic? How else has the pandemic impacted on their capability of imagining a new life without the departed?

## 2 METHODS

### 2.1 Participants

Twenty participants were recruited through snowball sampling (Goodman, 1961) in four heavily impacted towns in Lombardy (Italy), departing from two personal acquaintances of the interviewer who helped recruiting by word of mouth. Every participant contributed to put the researchers in contact with the next participant, based on a direct relationship or on a general acquaintance (only two participants were related as mother and daughter).

Table 1 reports participants' characteristics. Data collection lasted from April to August 2020, and, following the criteria for qualitative research, data collection and analysis of the interviews proceed simultaneously. Sampling ended once theoretical saturation was reached, that is the point at which gathering more data does not lead to more information related to the research questions (Flick, 2009).

**TABLE 1:** Participants' sociodemographic data

Factor	No respondents
Gender	
Female	15
Male	5
Mean age	40 y.o (SD = 13,9)
20–40	11
41–70	9
Ethnicity	
Caucasian	20
Mean decedent's age	72 y.o. (SD = 10,6)
50–70	8
71–90	12
Kinship	
Adult children	16
Grandchildren	3
Spouse	1
Cohabitating with decedent	
Yes	9
No	11 (4 in a retirement home)
Time elapsed since the death	
A month	11
Two months	6
Three months	3
Profession	
Unemployed	3
Housewife	2
Student	2
Employee (office worker)	4
Retired	2
Worker	5
Entrepreneur	1
Educator	1
Educational level	
Secondary school	4
High school	13
Bachelor's degree	2
Master degree	1

The only inclusion criteria were the following: (a) having lost a close relative or partner to COVID‐19; (b) being more than 18 years old and (c) residing in Lombardy. Potential participants were reached by telephone and gave their informed consent to take part in the study. The Ethical Committee of Psychological Research of the University of Padua approved the study.

### 2.2 Measures

Semistructured interviews were carried out through video calls to comply with the restrictions in place at the time. Interviews, although flexible, followed a common guide composed by eleven questions: Would you like to tell me about your loss? Where did your significant other die? Did you manage to take care of your significant other? If not, how did you manage to get information on his/her health condition? Did you manage to organize a funeral? If not, how does this make you feel? Is there anyone able to offer comfort and support? If yes, who are they and how do you stay in contact with them? If no, would you like to receive such support? Have you contacted a professional, or have you been contacted by a mental health service offered by your local administration? If no, would you like to?

### 2.3 Procedure

Participants who were interested in taking part in the study gave their consent to the participants contributing to the sampling procedure to give their telephonic contact to the interviewer who contacted them by telephone. Participants chose the place and time of the interview in order to respect their grieving needs (Bentley & O'Connor, 2014; Stroebe, Stroebe, & Schut, 2003). They participated in the video call from their homes and were encouraged to take their own time in talking about their experience. The interviewer is a doctor in psychology trained in conducting qualitative interviews. She shared with the participants her place of origin and experience of the COVID‐19 quarantine but did not report loss of a relative due to the disease. Sharing a common background—and in two cases also an acquaintance relationship—might have acted as a facilitator (Archibald & Munce, 2015) to make the participants more at ease, which is particularly important with the representatives of an enclosed and shocked community such as the one considered in our study. At the same time, the interviewer's professional point of view and the supervision she received on the part of one of the other authors with an established clinical and research experience allowed her to keep a superordinate perspective and comprehend a variety of experiences. The interviewer explicitly said to the participants that their own experience was relevant and that they had not to assume that the interviewer already knew their stories. This credulous approach was kept throughout the interview by posing questions to explore each answer without assuming that participant's meanings were the same of the interviewer.

The interviewer provided contacts of local associations and services that could provide free‐of‐charge support and counselling should they experience emotions of distress (e.g., after the interview). The average duration of the interviews was around 45 min (ranging from 28 to 106 min), and they were held in Italian, recorded and subsequently transcribed by the interviewer verbatim.

### 2.4 Data analysis

Clarke and Braun's (2018) process for conducting thematic analysis was followed, and the software ATLAS.ti8 was utilized to manage the data. Initial familiarization with the data and subsequent coding was carried out by two of the authors: one was also the interviewer; the other was a trained coder who supervised the open coding and contributed to the following steps in coding. Coders read and re‐read the data set until the depth and breadth of the content were familiar; memos and comments were also extensively used. In the second step, initial codes were generated from data; then, similar codes were put together in overarching themes, the relations between codes and formed themes were identified, and some themes and codes were discarded. The results were revised to evaluate if all the themes had sufficient supporting data with appropriate extracts and to judge if they met internal homogeneity and external heterogeneity criteria. Finally, themes were refined and named to indicate what topics they captured.

Coding procedures were continuously discussed with a third researcher, an expert in qualitative studies, which allowed the reassessment of themes and interpretation of the results until consensus was reached. Since the authors acknowledge their active role in identifying patterns and themes, selecting which ones are of interest to be reported to the readers, reflexivity was carefully sought through repeated comparisons and step‐by‐step discussions about alternative interpretations of the results between all the researchers. The study was reported according to the Consolidated Criteria for Reporting Qualitative Research (Tong, Sainsbury, & Craig, 2007).

## 3 FINDINGS

Four themes resulted from the thematic analysis, as reported below, together with some significant quotations.

### 3.1 Reconstructions of the loss experience

The first theme refers to participants' attempts to retrace the events that led to the loss. Generally, the beginning of participants' stories recounted how fever, fatigue and even shortness of breath among their relatives were not immediately identified as dangerous symptoms. However, soon after the first appearance of symptoms, family members had to be hospitalized quickly due to the sudden deterioration of their health conditions. Their death was mainly unexpected: nine of the decedents were described as having no previous illnesses and being in good health, whereas only a small number of patients (6 of 20) were described as fragile because of their age or general health state. Moreover, some participants were reassured that the patient's health was stable and reported an initial optimism that their loved ones would soon return home, until they received the death notification.

Once admitted to the hospital, families were prohibited from entering the hospital and reported difficulties being informed or updated on the patient's health condition because of the overcrowding of hospitals. On several occasions, they found no support or understanding from the health care staff, who were busy handling the extreme emergency situation and reported the impossibility to assist in person their family members as a great difficulty; only a small number, however, found health care professionals' (mainly nurses') availability to hold the telephone for a video call with the patient and recounted how this provided a momentary interruption of the profound anguish characterizing the hospitalization period:

Then again, [there were] a couple of very nice doctors, very human. But others were really the opposite … I'm telling you, they called me only a couple of times, one of which after 22/23 days that my father was intubated, telling me, ‘You get it that he is very serious, right?’ (P13, F, Daughter)

For some family members, even saying goodbye through telephone was not possible; this, and the impossibility to see the body in the hospital or before the burial, produced in some doubts about whether their relatives had been treated correctly:

Maybe [I am afraid to] discover that my husband had suffered because no one had been able to do their duty as they should have. (P12, F, Wife)

In two cases, participants were not allowed to hospitalize their relatives because of their age and, consequently, experienced the death of their loved one in the home: in one of these cases, the patient was administered palliative care and the participant described the death as the end of ‘a Calvary’. This common expression in the Catholic cultures refers to the place of Jesus' crucifixion and, such as the word ‘excruciating’, it implies an extremely painful emotional and physical experience

### 3.2 Responses to grief

Several interviewees (11 of 20) reported common responses to grief such as shock, apathy, ruminative thinking, concentration problems, and physical issues (poor appetite, weight loss and sleep disturbances)

Honestly, I was distressed every time the phone rang; I still wake up at night when I hear the phone ring. I also find it hard to concentrate. I think it's a phase of pain, I don't know. (P7, F, Daughter)

Another common experience was a sense of void left by the departed, not only in terms of a radical change in everyday life habits but also concerning the loss of an important resource the mourner could rely on, or even a part of one's self. Equally noted was the regret for having underestimated the virus' lethality: in some cases, participants were also afraid of having infected the deceased. Other participants felt guilty and responsible for their loved one's death because they feared that they had contracted the virus at the hospital where they had taken them

Then you tell to yourself… If I had not taken him to the hospital […] He caught the virus in the hospital and now I also have this regret. (P3, M, Son)

Several participants (13 of 20) reported difficulties realizing the death of their loved one had actually occurred. This feeling was reinforced in these mourners due to the impossibility of seeing the body or having a funeral. The lack of a ritual was described as ‘unnatural’ and ‘nightmarish’: mourners talked about a ‘violation of grief’, which led to a deep suffering. Because of the sudden deployment of the events, some participants were even unsure whether the remains returned to them were actually those of their loved ones:

He came back from the hospital and [the coffin] was, rightly so, closed and therefore not being able to see him, even if dead, [to] still be able to see his face, his expression, to see his body […] you don't realize it. (P5, F, Granddaughter)

The uncertainty and fear of the contagion also influenced those actions commonly performed after a loss and that are functional to the loss elaboration. For example, one participant said she had been unable to even touch the deceased's possessions returned from the hospital, fearing they could still be infected. Six participants were able to organize alternative rituals to make up for the lack of a funeral – for example, by having the hearse pass the house before heading to the cemetery.

So luckily, they gave us this way of, of taking him home with the hearse, carrying him around our house, getting to the cemetery and giving him a last farewell with the ceremony, with the priest, me, my mom and my sister. Let's say that we had this luck, while unfortunately other people did not. (P2, M, Son)

Anger characterized the stories of six participants who had lost a younger relative (between 51 and 60 years old) and had a strong difficulty accepting the death, reporting that they had difficulties crying and mourning and declaring they were convinced their loved ones would still be alive if they had not been hospitalized:

They stole him [the ill father] violently from me; he didn't deserve it, as none of these victims deserved it. My anger is they [doctors] claimed they were too old; this is what triggers my anger. (P3, M, Son)

These participants reported a strong willingness to find justice and, if necessary, to associate in groups and denounce institutions by reporting their own experience. Some of these participants put the blame on the doctors for having misdiagnosed the patient, suggested hospitalization or having provided improper care. Eleven participants blamed the slowness with which the administrations acted and complained that the government had abandoned them and treated their loved ones as expendable. Of these, five participants felt alone in their grief and abandoned by their families: the general lockdown or, in some cases, the necessity to quarantine resulted in the death of a loved one and cancelled any possibility for the person to access family members for comfort.

Ok, I am awfully angry at the region's administration, because I am convinced that if they had closed everything immediately, as it was in their ability to do, maybe my dad would have died anyway, but of over 30 thousand people in Lombardy, many of these would not. I repeat, maybe my dad would have, but […] Tomorrow I'm going to file a complaint. (P13, F, Daughter)

On a different note, five participants who had lost a senior relative, such as a grandparent or relative with previous illness, could accept what had happened and did not feel responsible for the death. They acknowledged that their loss had occurred under unexpected circumstances, understood that the restrictions were necessary and had been focusing, instead, on the necessity to warn others of the virus' lethality and to raise awareness about the necessity of adopting the correct behaviors:

Now I do not think that getting angry has … has great sense, or maybe I am just hoping that … people are more sensitized to prevent [the virus] from spreading. (P1, F, Granddaughter)

At this moment our mind remembers and remembers very well, so we must not allow this thing to slip away in a drawer; we must remember it well and pay attention. I see that people who have not been hit by it are not cautious. (P16, F, Daughter)

The need to testify to their own experience holds a different nuance of meaning than denouncing, as it is linked to the necessity of preserving the memory of the events, preventing them from happening again (especially by warning those in other regions still untouched by the virus when the interviews were held) and keeping the memory of loved ones alive. For others, acceptance meant acknowledging the possibility of at least saying goodbye to their loved ones and understanding there was nothing else they could do. Because the entire country was versed in the same situation, two participants explained that their loss was not individual but somehow shared; thus, this helped them feel less alone in their grief.

Let's say that knowing, knowing that in any case I am not the only person in this situation helps a little, no? I mean, I don't want to wallow in my pain because I'm the most unlucky in the world. (P1, F, Granddaughter)

The few participants who were grandchildren of the dead persons coped with the loss by supporting their own parents grieving, and this became a priority that could overshadow their own sufferance.

I think of my mother who has lost her mother, excuse the pun. And I think I have to support her as much as possible because she couldn't even say hello or see her [own mother] from a hospital window. Nothing at all. […] my brother is also there but we have to stay close to her because it is something that you easily accept. (P1, F, Grandson)

### 3.3 Personal resources and search for support

All participants were left mourning during the early stages of the pandemic: because of the social restrictions imposed by the lockdown and, in some cases, their own quarantine, participants sought out and obtained support for their loss mainly through telephone/video calls or messages. Despite the severe limitations they were, overall, able to find satisfactory support from their social network (families and friends). Social networks represented an important resource; in some cases, contact with friends even intensified thanks to social media, which allowed interviewees to feel closer to others and feel the support of the entire community.

As a matter of fact they [my friends] call me more than before to keep me up and try to get me through this bad time. They want the positivity to come back. (P6, F, Daughter)

Nevertheless, an interviewee pointed out the limitations of support offered mainly online in the long term: continuous online contact with people who tried to virtually support the bereaved person ended up weighing down the participant, who would have preferred to go out and focus on something other than her loss.

How do you support if you can't hug? If you can't have a real relationship? Let's face it, making a video call is nothing compared to talking to the person in front of you, having the person at 50 cm from you. (P5, F, Granddaughter)

The availability of virtual means to keep connected does not, however, offer the certainty of social connectedness. Two participants declared to not have searched for help in their social relationships and withdrew from them or avoided the discussion over the death: ‘Often I find myself surrounded by people who annoy me even, you know? Because the thoughts are always there’. (P16, F, Granddaughter)

This could be even truer for the provision of professional support. The majority of interviewees (12 of 20) had not looked for professional help, either online or offline because they did not trust the mental health services or were confident they could succeed on their own.

He [the doctor] would send me to a colleague of his who was doing relaxation sessions as well as psychotherapy. All for free here at the Local Health Services. I said that right now I don't want it, I don't feel the need. I want to see if I can come out of it on my own. I have always come out of my problems by myself. (P12, F, Wife)

Some participants showed to resolve to spirituality and religion to feel heartened despite the conditions in which the loss occurred.

My hope is that I will see him on the other side. This is the thing that cheers me up a bit when I am gripped by anguish. Otherwise, it would be … those who do not believe, I wonder how they do it, for me it would mean desperation. I couldn't do it. (P12, F, Wife)

### 3.4 Looking forward

Within this theme, participants reported their expectations, hopes, wishes and needs regarding their future after the loss. The main issue that mourners looked forward to was a proper funeral for their loved one, where it would have been possible to invite all of their loved ones and receive physical comfort through hugging – that is, physical contact with others was an important and neglected factor. Funerals were also indicated as necessary to realize the factuality of the death, as well as an opportunity to carry out the ceremony in the manner desired by the deceased, who were often very religious, and in doing so to maintain the bond with the deceased.

Those who stated they would not look forward to a funeral explained either that they were non‐religious or that they preferred to avoid other events that could promote the spread of the virus:

To tell you the truth, I would prefer not to [have a funeral] because in this moment where they [administrations] do not know how to manage the situation, the less you meet the better. (P6, M, Son)

Some of them also reported the need for time and space to process the loss, whereas others wanted to focus on ways to restart – for example, by finding a new job:

In a nutshell, what I would like is to be able to restart; I do not say to be active again … gyms are not accessible and that's ok, I do it from home, but to be able to have a job and be able to have a life that can give me a future. (P9, F, Daughter)

Participants whose narrations were characterized by the anger for administrations' and governments' mismanagement asked for justice and punishment for the guilty; differently, those who had already started a sensemaking process called for a drastic change in the framework of administrations that would restore focus on the citizens' health and not on economic interests, as well as move toward collaboration, mutual care and compassion between citizens to conteract the devastating effects of the pandemic.

Let's put those two [local politicians] in jail. Okay, we can't resurrect the dead, but those two [should] go to jail because they have to pay a lot, pay in the sense… We don't want money. And they have to never be re‐elected, never again. Whoever made a mistake must pay. (P11, F, Daughter)

I would like people, I for one, after this tragedy, to think a little less about the economy and a little more about loving each other; otherwise this tragedy will have taught us nothing, leaving us with only our losses to mourn and nothing else. (P3, M, Son)

## 4 DISCUSSION

This study explored the experiences of the bereaved following the death of a loved one to COVID‐19. Such investigations from more heavily impacted areas are pivotal for shedding light on the experience of traumatic bereavement during the COVID‐19 pandemic. The unpreparedness on the part of local administrations and healthcare system (Bosa et al., 2021) to contain the infection and manage the health emergency may have further adversely impacted those who lost a significant person in their lives.

Indeed, participants' experiences were characterized by uncertainty throughout the process that led to the deaths of their loved ones. Confusion about how to determine a COVID‐19 case, which symptoms to look for or the real risk of a widespread diffusion of the virus in the community was still high, even among doctors. Moreover, the only way for participants to communicate with their loved ones in the hospital was through nurses holding the phone close to the bed. This example of the care and compassion of frontline health care workers likely made a difference in mourners' capability to make sense of their loss (Wakam, Montgomery, Biesterveld, & Brown, 2020). Communication in end‐of‐life care is of critical importance, and health professionals should be educated to provide this type of support, which is pivotal for both the patients preparing for their own departure and their family members (Cipolletta & Reggiani, 2021; Ersek et al., 2021).

The COVID‐19 mourners included in this study had all experienced a recent loss (i.e., less than 6 months) and felt regret, bewilderment and shock, which are characteristic of the first months of mourning a traumatic loss (Ross, Kõlves, Kunde, & De Leo, 2018). Experiencing loss during the COVID‐19 pandemic can hinder meaning‐making for grieving families (Eisma & Tamminga, 2020): confinement to home, financial precarity and constant worrying about personal or others' health contribute to mourners' struggle to undertake active actions such as obtaining practical and emotional support and attending to life changes (Neimeyer & Burke, 2017), which are pivotal for meaning‐making and post‐traumatic growth (Milman et al., 2017; Neimeyer, 2019).

The lack of a funeral was considered significant by mourners, who longed for the hugs of family members and the opportunity to publicly experience their pain, although some of the participants' narrations support Burrell and Selman's (2020) claim that alternative ways of commemorating the deceased in a personal and meaningful way may compensate for the lack of attendance at a funeral; this obstacle to the farewell is a great lack and professionals should base their interventions on the possibility of constructing a sense for what happened, possibly through the creation of meaningful rituals to replace the lost ones (Borghi & Menichetti, 2021) such as lighting a candle, holding a ceremony outdoor (e.g., in a private garden) or collecting songs, photos and stories linked to the departed (for a review, see Burrell & Selman, 2020).

Among all the participants, those who struggled more to give meaning to their loss were those mourners who believed their loved ones departed prematurely, or unjustly; they felt angry toward professionals for not having avoided a preventable death (Ahmadi & Ramezani, 2020) and put blame on administrations in order to make sense of their loss (Carr et al., 2020; Neimeyer, 2001), while denying the emotions linked to the loved one's death. With the COVID‐19 pandemic being a global‐scale event, mourners are constantly reminded of the circumstances of their loved ones' deaths; for those struggling to accept their loss, it could become tempting to concentrate all their energies on associating in groups and to pour their anger into the pursuit of justice rather than begin the process of accepting the loss. The disappointment toward inadequate administrations, which was only briefly described in studies reporting practitioners' experience (Alizadeh et al., 2020; Dhairyawan, 2021), was observed consistently throughout the experiences of several participants and took the form of open conflict with the governance. This attitude could be attributed to the specific way in which the Italian population relates to politics (Capano, 2020) and often mistrusts healthcare services (Cipolletta & Reggiani, 2021) and is a factor that should be considered for further studies: its presence should be verified in different Western cultural contexts because this study shows how it could significantly affect the ability to adapt to the pandemic scenario, as well as to the loss.

The small number of participants able to accept the COVID‐19 death in the range of less than 6 months leaves hope that a larger number will be able to do so after 12 or 24 months, as has been observed in previous studies (Entilli, Ross, De Leo, Cipolletta, & Kõlves, 2021). Longitudinal studies are needed to test this hypothesis.

The experience of loss may also vary depending on the type of relationship with the deceased (Cerel, McIntosh, Neimeyer, Maple, & Marshall, 2014), for example, grandchildren coped with the loss of their grandparent by supporting their own parents grieving. In the present study, only four participants had a kinship other than being children of the deceased, and this dimension was therefore not extensively explored. Gender differences could not be extensively explored either because most of the participants were women. Nevertheless, results did not show substantial differences in men's and women's responses to the loss (e.g., anger, resignation and hostility).

The results of the present study shed light on mourners' recourses to cope with loss. As already observed in the literature (Coppola, Rania, Parisi, & Lagomarsino, 2021), mourners have shown in some cases to rely on personal faith as a strategy to cope with the traumatic conditions in which they have lost their loved one; this type of resource providing comfort, support and meaning, especially in the Italian context, must be taken into consideration by mental health professionals. Most of the respondents did not show interest in seeking traditional psychotherapy close to their loss but could be looking for support to process emotions. Timely, short‐term and easily accessible means such as live‐chats or online groups may be useful to offer initial emotional buffer and to lay the groundwork for the request of a professional support, by offering a place where to break the silence around death (Miles & Corr, 2017) and also by providing the information needed to access it (Cipolletta et al., 2021). These interventions might represent a first step or an adjunct to face‐to‐face interventions aimed at legitimizing emotions while also promoting self‐efficacy and avoid social withdrawal (Lissoni et al., 2020) and fostering meaning‐making.

In the present study, the provision of support was aided by the technological tools to compensate for social distancing, as already pointed out in previous studies (Goel & Gupta, 2020; Testoni et al., 2021). However, participants indicated virtual support as a valid solution only for the short term. In the long term, a more extensive action involving the mobilization of compassionate communities is needed to cope with the traumatic loss (Abel & Taubert, 2020): emotional closeness might be maintained through compassion and mutual support, by means not only of the new technologies but also of bereavement meetings and cafes.

The current study is limited to the bereavement experience of Italian COVID‐19 mourners and, its results might not reflect the experiences of mourners belonging to other cultures or vulnerable groups (e.g., ethnic minorities or individuals with a disability) who might struggle with different problems. Moreover, the procedure of sampling through the word of mouth might have favoured the participation of those who were more willing of sharing their experience, that in this study were women and adult children of the dead person. Due to the composition of the sample, some dimensions such as gender differences or the type of bond with the dead person have not been extensively analysed and should be the subject of further study.

## 5 CONCLUSIONS

At the time, the present article was written, other authors (Boelen et al., 2020; Eisma et al., 2020; Kokou‐Kpolou et al., 2020; Masiero, Mazzocco, Harnois, Cropley, & Pravettoni, 2020; Mortazavi, Assari, Alimohamadi, Rafiee, & Shati, 2020; Wallace et al., 2020; Zhai & Du, 2020) have only anticipated possible grief outcomes based on previous experience with other traumatic losses. What has not been found or anticipated in literature regarding COVID‐19 deaths is mourners' need to witness and denounce a situation perceived as unjust (Stroebe & Schut, 2020) as well as the more functional will to warn others and avoid recurrence of such a situation, which were the main coping strategies pointed out by the present study.

The observed wariness or direct hostility of mourners toward mental health support may have implications for treatment. Professionals approaching COVID‐19 mourners should be aware that their patients could be extremely disheartened by health services, and as a result, their alliance or compliance may already be fragile (Gravante & Poma, 2021; Testoni et al., 2021). Understanding this attitude rather than getting into conflict with it may help to construct a cooperative enterprise where the mourning person and the professional work together for a common aim. This solution is in line with the one found by those participants who used their own, dramatic, experience as a turning point to seek a change of focus from economic benefits to citizen support, responsibility and reciprocal care: they stressed the necessity to switch to a different policy where the person, rather than the economy, is the focus (Cipolletta & Ortu, 2021).

As Stroebe and Schut (2020) highlighted, there is still relatively little consideration of positive or compensatory processes for possible alleviation of grief in COVID‐19 mourners. The ability to find a silver lining is pivotal to access meaning reconstruction (Holland, Currier, & Neimeyer, 2006). Mourners felt relieved thinking their experience was of shared grief with an extended group of other people. Socially shared grief could be a key binding factor for the ability to make sense of the loss, and it could be a good starting point for clinicians intending to foster meaning‐making. The results of the present study provide a useful framework for looking further into appraisal processes and their associations with adaptation to loss in pandemic circumstances.

## CONFLICT OF INTEREST

Authors declare that they have no conflict of interest.

## Supporting information

Supporting Information

## Data Availability

The data that support the findings of this study are available on request from the corresponding author. The data are not publicly available due to privacy or ethical restrictions.
