# Supplementary material for: Uncertainty, shock and anger: Recent loss experiences of first‐wave COVID‐19 pandemic in Italy
Source: J Community Appl Soc Psychol. 2022 Feb 13;32(5):983–97. doi: 10.1002/casp.2604 (PMC9083240; doi:10.1002/casp.2604)
Supplement: Supplementary file 1 — Supporting Information [file CASP-32-983-s001.pptx]

## Slide 1
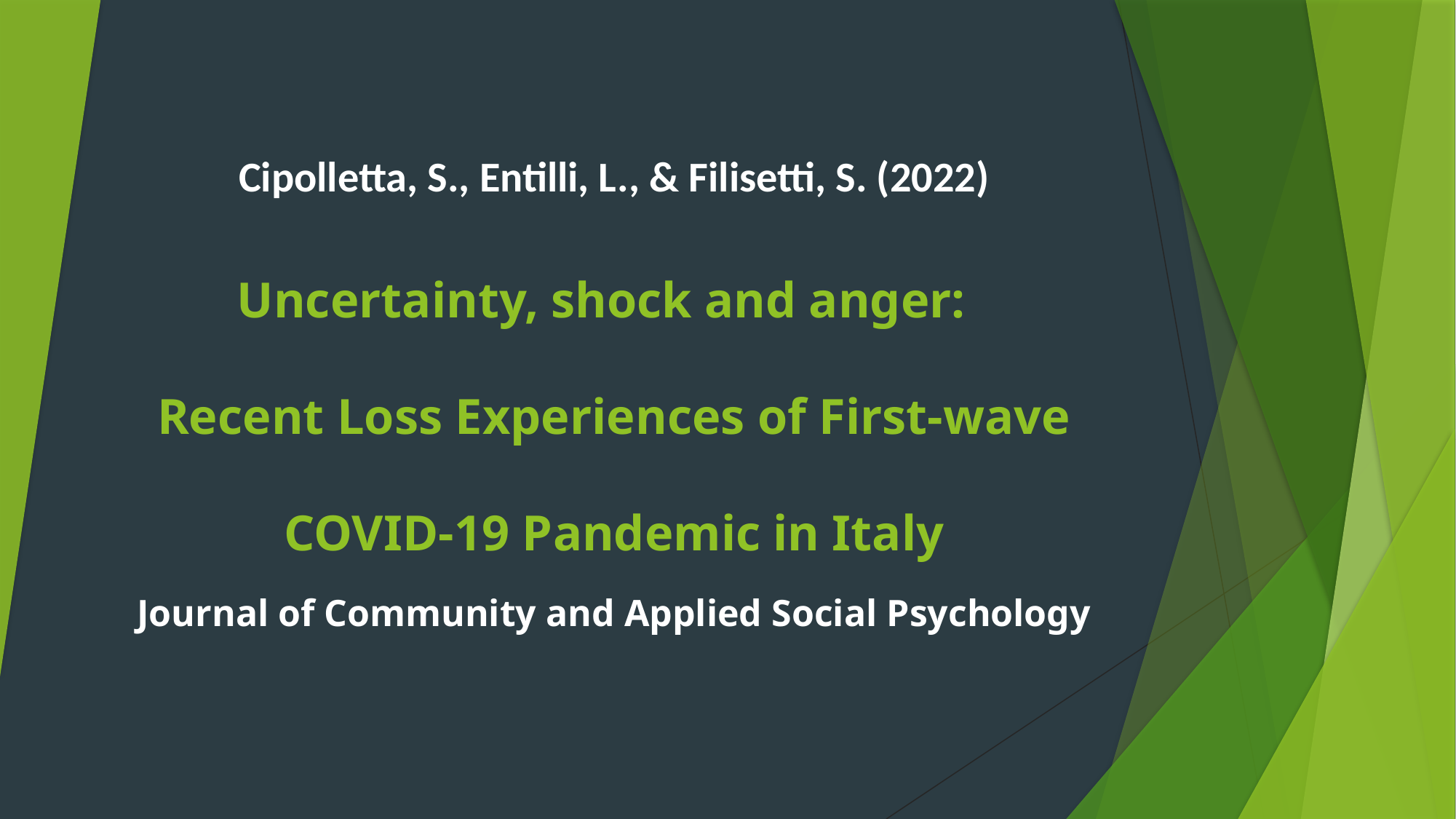

Cipolletta, S., Entilli, L., & Filisetti, S. (2022)
Uncertainty, shock and anger:
Recent Loss Experiences of First-wave COVID-19 Pandemic in Italy
#
Journal of Community and Applied Social Psychology

## Slide 2
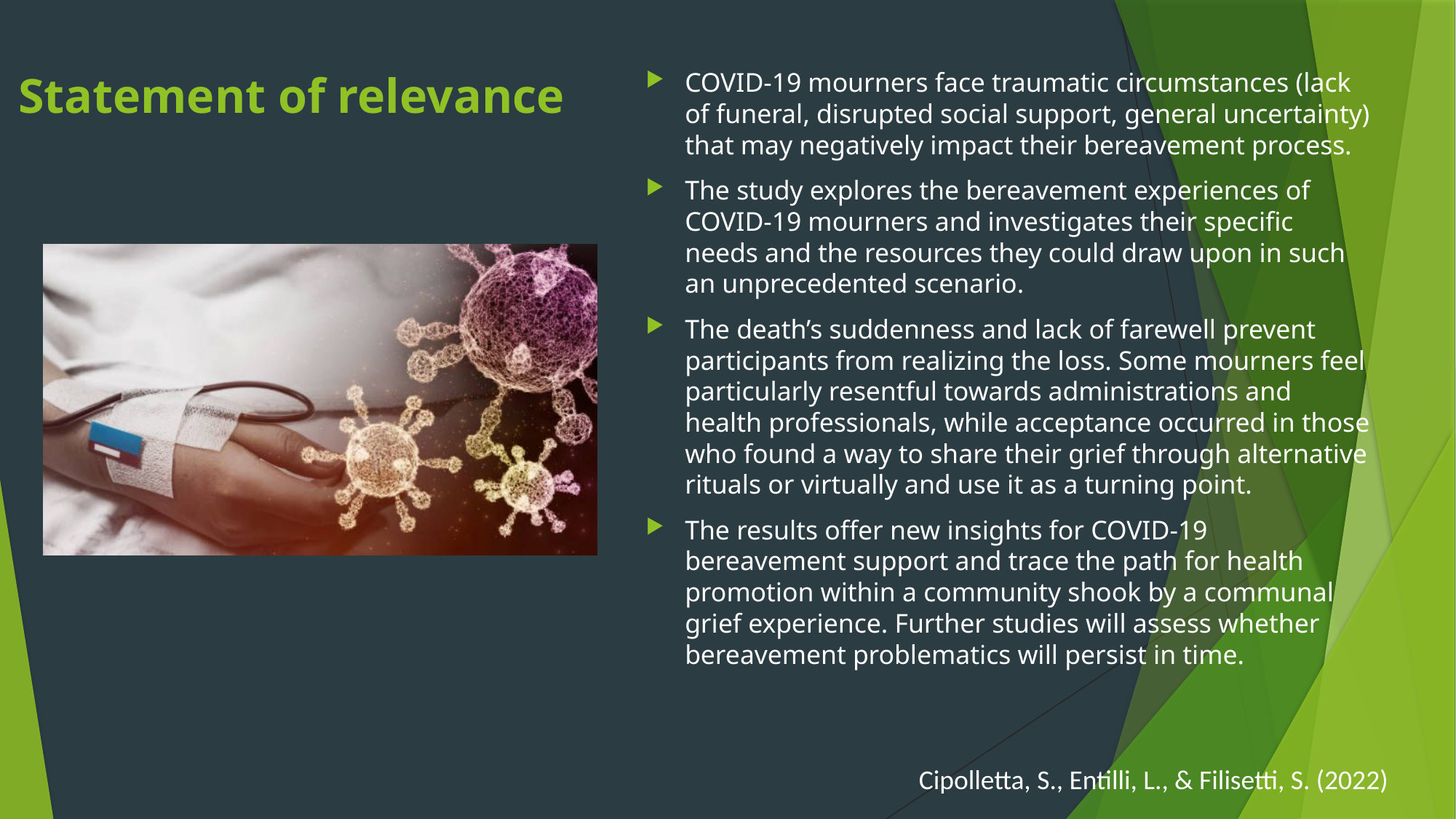

# Statement of relevance
COVID-19 mourners face traumatic circumstances (lack of funeral, disrupted social support, general uncertainty) that may negatively impact their bereavement process.
The study explores the bereavement experiences of COVID-19 mourners and investigates their specific needs and the resources they could draw upon in such an unprecedented scenario.
The death’s suddenness and lack of farewell prevent participants from realizing the loss. Some mourners feel particularly resentful towards administrations and health professionals, while acceptance occurred in those who found a way to share their grief through alternative rituals or virtually and use it as a turning point.
The results offer new insights for COVID-19 bereavement support and trace the path for health promotion within a community shook by a communal grief experience. Further studies will assess whether bereavement problematics will persist in time.
Cipolletta, S., Entilli, L., & Filisetti, S. (2022)

## Slide 3
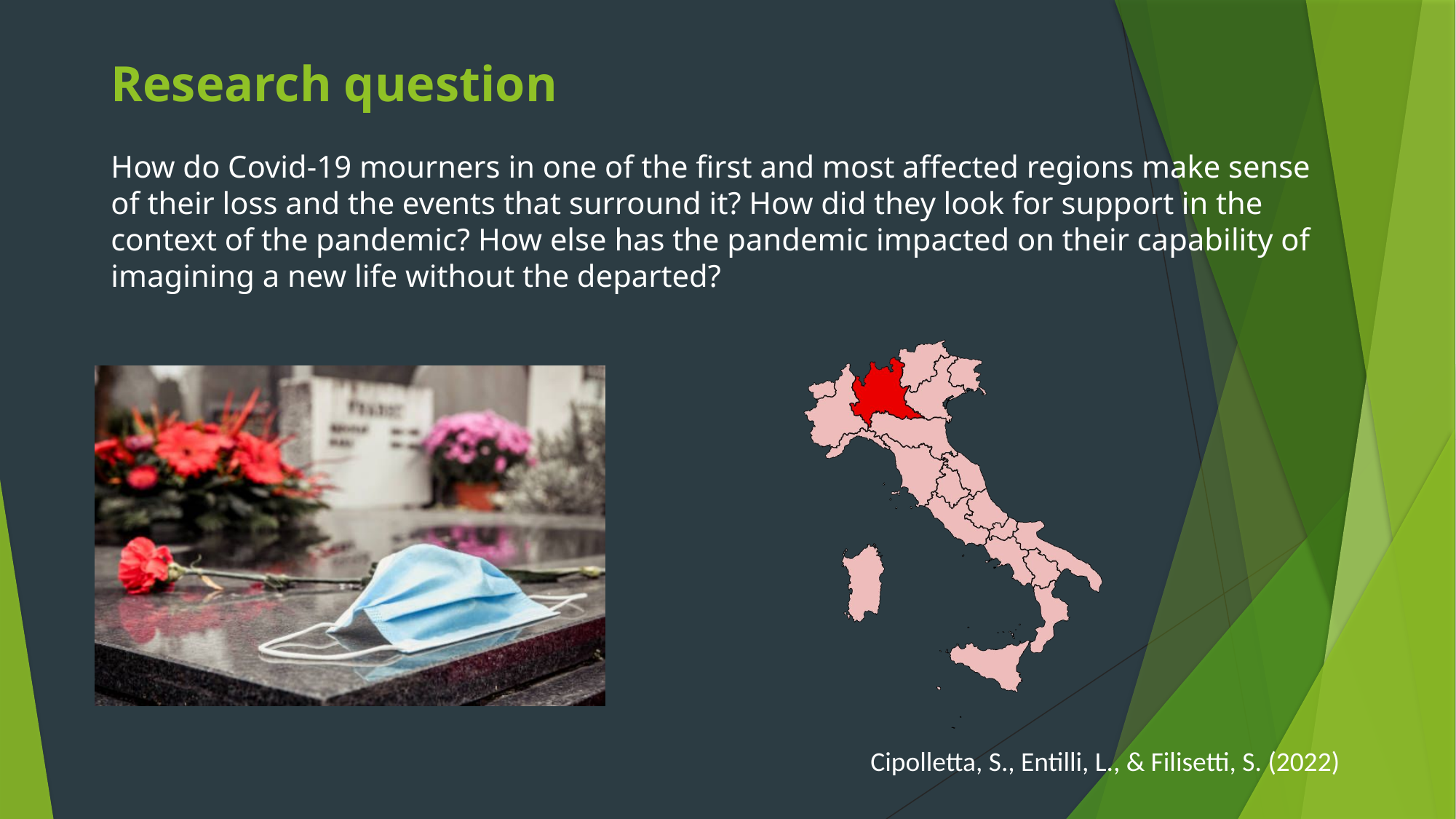

Research question
How do Covid-19 mourners in one of the first and most affected regions make sense of their loss and the events that surround it? How did they look for support in the context of the pandemic? How else has the pandemic impacted on their capability of imagining a new life without the departed?
Cipolletta, S., Entilli, L., & Filisetti, S. (2022)

## Slide 4
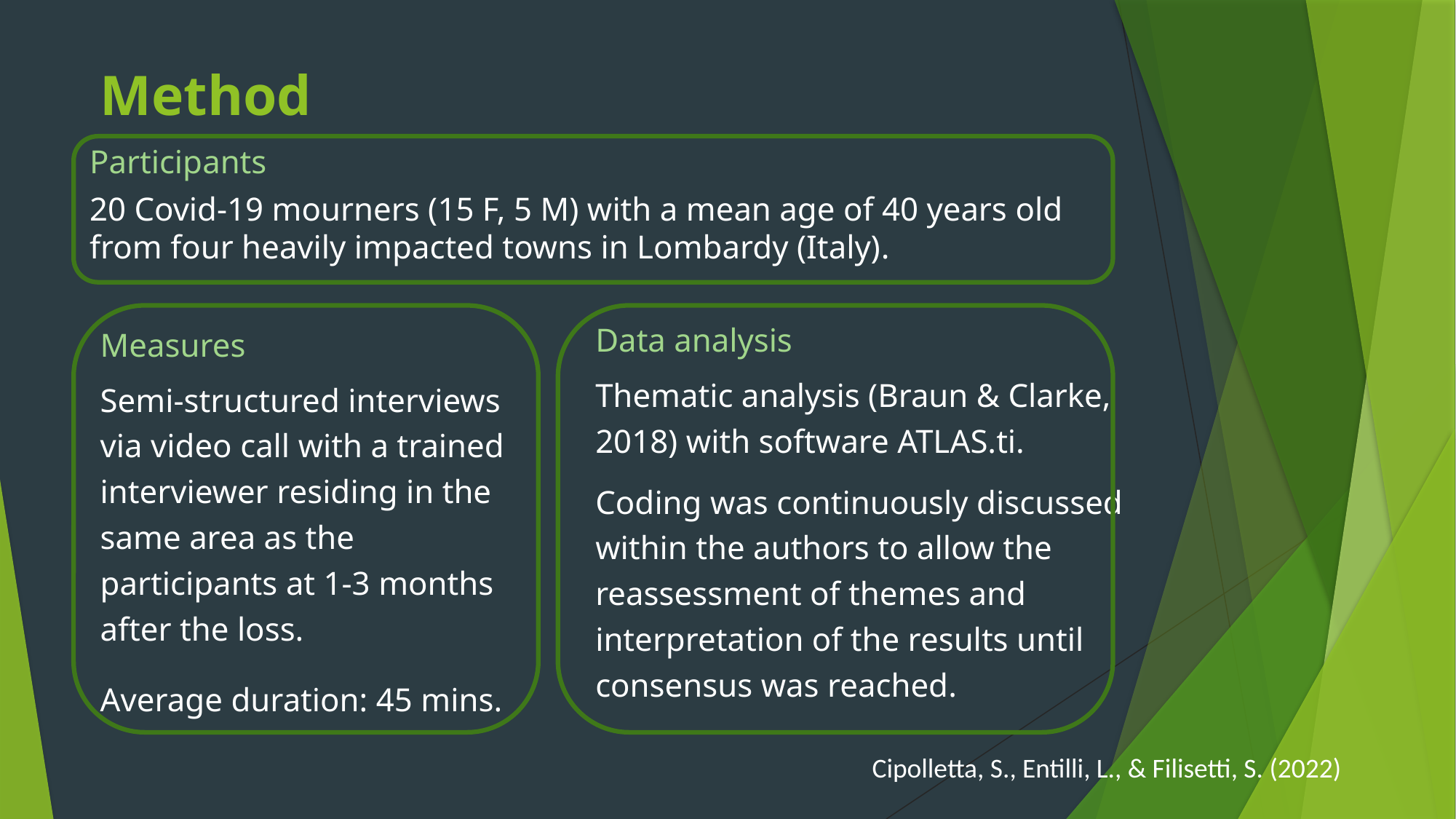

# Method
Participants
20 Covid-19 mourners (15 F, 5 M) with a mean age of 40 years old from four heavily impacted towns in Lombardy (Italy).
Measures
Semi-structured interviews via video call with a trained interviewer residing in the same area as the participants at 1-3 months after the loss.
Average duration: 45 mins.
Data analysis
Thematic analysis (Braun & Clarke, 2018) with software ATLAS.ti.
Coding was continuously discussed within the authors to allow the reassessment of themes and interpretation of the results until consensus was reached.
Cipolletta, S., Entilli, L., & Filisetti, S. (2022)

## Slide 5
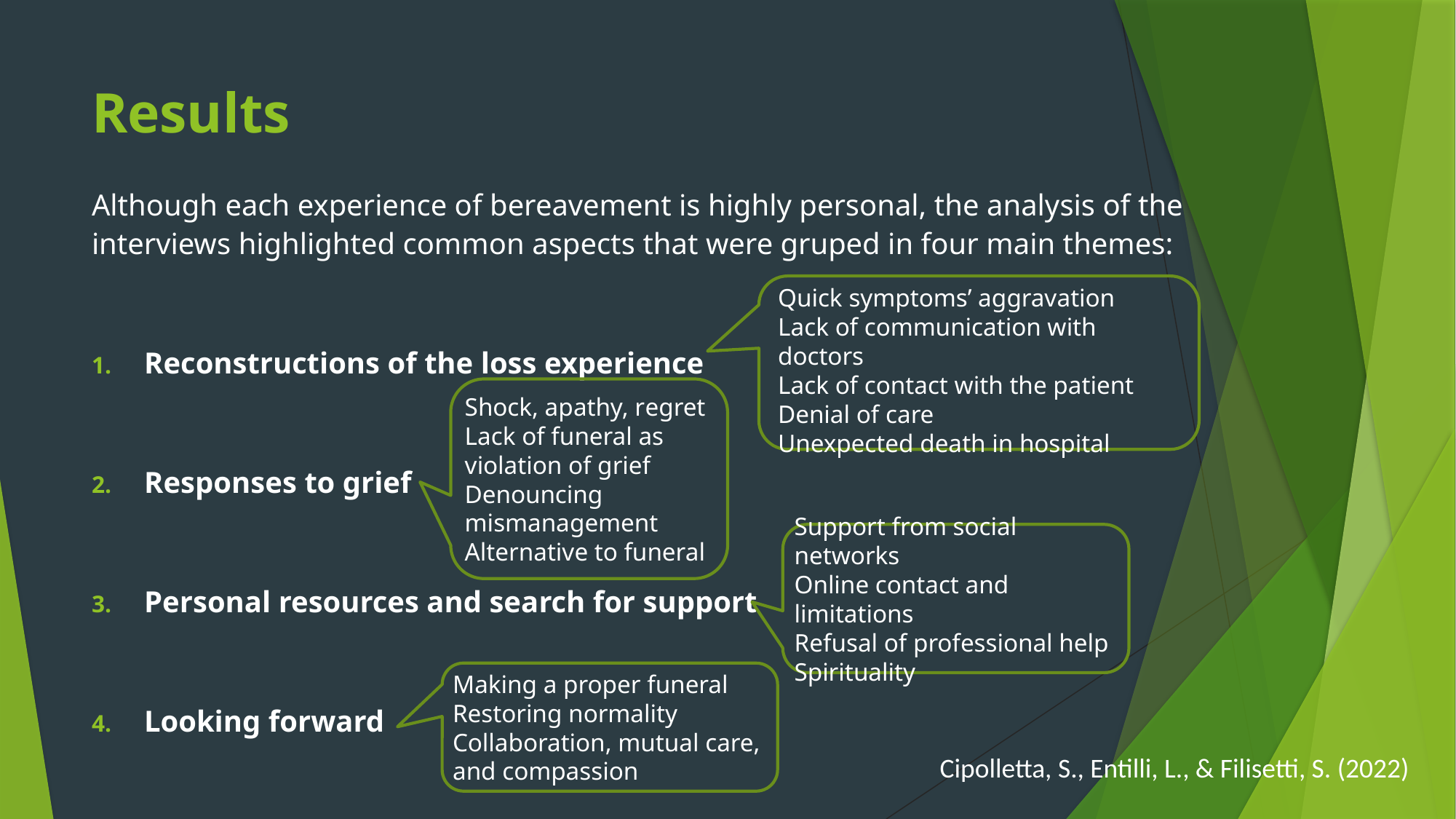

# Results
Although each experience of bereavement is highly personal, the analysis of the interviews highlighted common aspects that were gruped in four main themes:
Reconstructions of the loss experience
Responses to grief
Personal resources and search for support
Looking forward
Quick symptoms’ aggravation
Lack of communication with doctors
Lack of contact with the patient
Denial of care
Unexpected death in hospital
Shock, apathy, regret
Lack of funeral as violation of grief
Denouncing mismanagement
Alternative to funeral
Support from social networks
Online contact and limitations
Refusal of professional help
Spirituality
Making a proper funeral
Restoring normality
Collaboration, mutual care, and compassion
Cipolletta, S., Entilli, L., & Filisetti, S. (2022)

## Slide 6
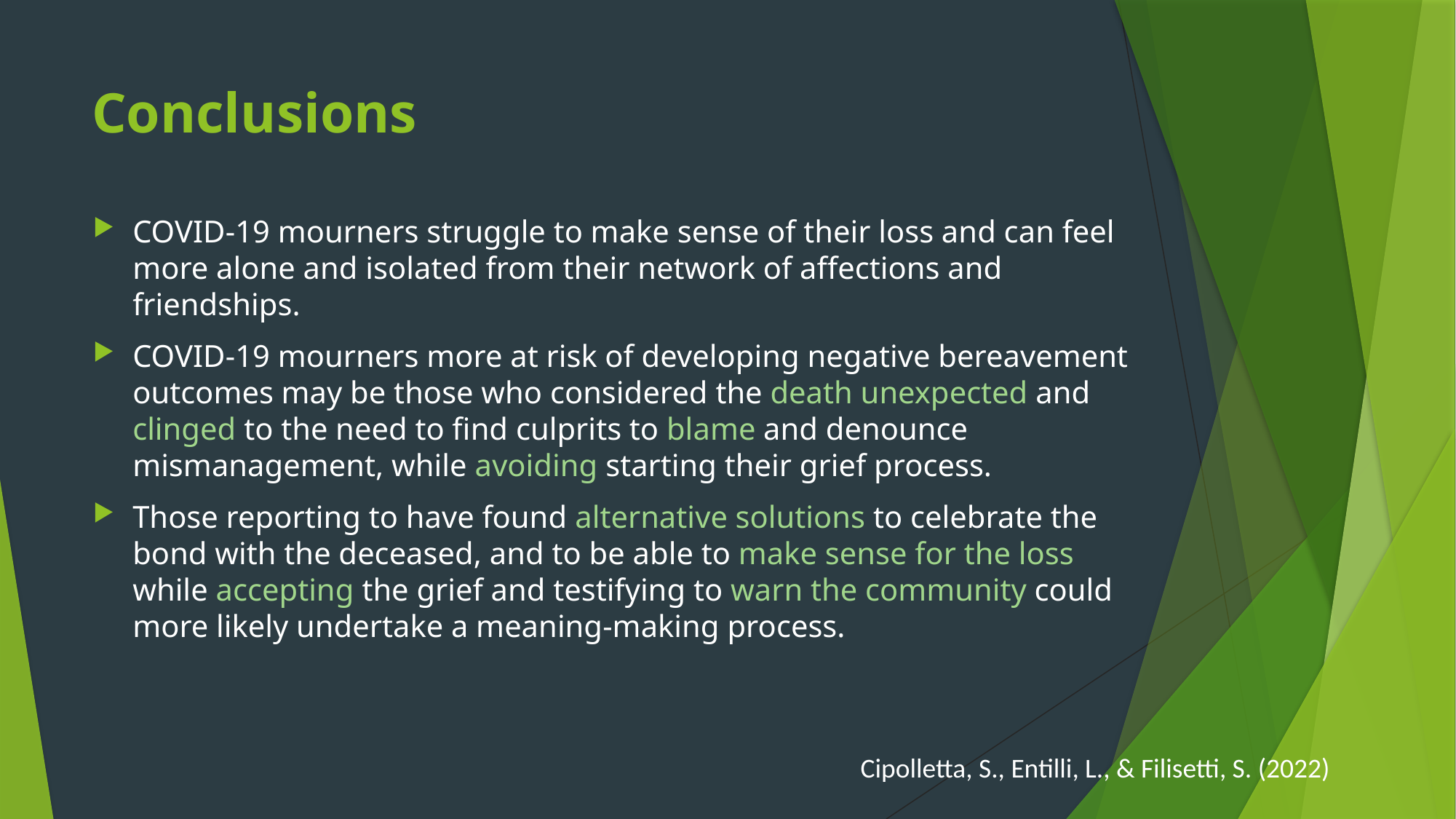

# Conclusions
COVID-19 mourners struggle to make sense of their loss and can feel more alone and isolated from their network of affections and friendships.
COVID-19 mourners more at risk of developing negative bereavement outcomes may be those who considered the death unexpected and clinged to the need to find culprits to blame and denounce mismanagement, while avoiding starting their grief process.
Those reporting to have found alternative solutions to celebrate the bond with the deceased, and to be able to make sense for the loss while accepting the grief and testifying to warn the community could more likely undertake a meaning-making process.
Cipolletta, S., Entilli, L., & Filisetti, S. (2022)
